# Effect of lead exposure from electronic waste on haemoglobin synthesis in children

**DOI:** 10.1007/s00420-020-01619-1

**Published:** 2021-01-20

**Authors:** Hongwu Wang, Peng Huang, Ruibiao Zhang, Xueyong Feng, Qiulin Tang, Sixi Liu, Feiqiu Wen, Li Zeng, Yufeng Liu, Tianyou Wang, Lian Ma

**Affiliations:** 1 Department of Hematology and Oncology, Shenzhen Children’s Hospital of China Medical University, Shenzhen, 518038 China; 2 Department of Pediatrics, The Second Affiliated Hospital of Shantou University Medical College, Shantou, 515041 China; 3 Shenzhen Public Service Platform of Molecular Medicine in Pediatric Hematology and Oncology, Shenzhen, 518000 China; 4 Beijing Children’s Hospital, Beijing, 100045 China; 5 Children’s Hospital of the First Affiliated Hospital of Zhengzhou University, Zhengzhou, 450000 China; 6 Department of Clinical Laboratory of the First Affiliated Hospital of Zhengzhou University, Zhengzhou, 450000 China; 7 Department of Pediatrics, Third People’s Hospital of Huizhou, Affiliated Huizhou Hospital of Guangzhou Medical University, Guangdong, 516002 China

**Keywords:** E-waste, Blood lead, Haemoglobin, Anaemia, Preschool children

## Abstract

**Background:**

Primitive electronic waste (e-waste) recycling is ongoing in Guiyu, so toxic heavy metals may continue to threaten the health of children in the area.

**Objective:**

This study primarily aimed to evaluate the effect of e-waste exposure on haemoglobin (Hb) synthesis in preschool children.

**Methods:**

Medical examinations were conducted with the permission of children’s guardians and the approval of the Ethics Committee of the Medical College of Shantou University. This study recruited 224 children (aged 3–6 years, exposed group) who lived in Guiyu and 204 children (aged 3–6 years, control group) who lived in a town free of e-waste pollution. Blood levels of lead, Hb, ferritin, folate and vitamin B_12_ were tested in all children. Furthermore, all children were assessed for thalassemia, and their parents were asked to fill in questionnaires.

**Results:**

There were no significant differences in the level of ferritin, folate, or vitamin B_12_ between the exposed and control groups (*P* > 0.05). No children were identified as having thalassemia in all study participants. Blood lead level (BLL) and the risk of children with BLL ≥ 10 µg/dL in the exposed group were significantly higher than those in the control group (all *P* < 0.01). Three subgroups of each group were created according to BLL (Group A: < 5.0 µg/dL; Group B: 5.0–9.9 µg/dL; Group C: ≥ 10.0 µg/dL). Hb level decreased with elevated BLL in the exposed group (*P* = 0.03), but not in the control group (*P* = 0.14). Hb levels in group B and group C were also significantly lower in the exposed group than in the control group (Group B: 122.6 ± 9.5 g/L versus 125.8 ± 8.2 g/L, *P* = 0.01; Group C: 120.3 ± 7.3 g/L versus 123.6 ± 8.3 g/L, *P* = 0.03). In addition, the prevalence of anaemia associated with BLLs above 10 µg/dL and between 5.0 and 9.9 µg/dL were both significantly higher in the exposed group than in the control group (4.0% vs. 0.5%, 5.4% vs. 1.5%, respectively, both *P* < 0.05).

**Conclusion:**

Lead exposure more significantly inhibits Hb synthesis in children who live in e-waste dismantling areas than in those who live in non-e-waste dismantling areas. Other toxins released from e-waste may also contribute to the inhibition of Hb synthesis and may lead to anaemia in local children. Further investigations are needed to provide evidence for the development of relevant protective measures.

## Introduction

Childhood is a period of rapid growth and development of all the organs and systems of the body. In addition to genetic factors that may lead to disorders of the blood system, the lack of various haematopoietic substances, such as dietary nutrients, proteins, and vitamins, can seriously impact it. Haemoglobin synthesis requires the coordinated production of haeme and globin. Haeme is the prosthetic group that mediates reversible binding of oxygen by haemoglobin. Globin is the protein that surrounds and protects the haeme molecule. Haeme or globin synthesis disorders can cause haemoglobin synthesis to decline. Moreover, the toxic effects of harmful environmental substances may inhibit the synthesis of normal haemoglobin (Hb) in children and even lead to anaemia. Anaemia not only affects children’s physical and mental development but also makes children susceptible to some diseases that can pose a great threat to their health and even endanger their lives. According to a survey of anaemia in Guangdong, China, the prevalence of anaemia among children aged 3–6 years in underdeveloped rural areas in Guangdong is 10.73% (Xiao-feng et al. 2015). There are many causes of anaemia in children. The common causes are iron deficiency, nutritional megaloblastic and thalassemia.

Except for the most common causes of anaemia in children, lead is recognized as one of the main environmental toxins that has the ability to inhibit Hb synthesis. The mechanism for this inhibition is as follows: lead can inhibit aminoethyl propionate dehydrase (ALAD), which hinders the conversion of aminoethyl propionate (ALA) into protoporphyrin and reduces Hb synthesis. In addition, lead can inhibit iron-chelating enzymes and prevent protoporphyrin and divalent iron from synthesizing haeme. Under the influence of lead, the conformation of Hb changes, and the sensitivity of Hb to proteolytic enzymes increases, thus accelerating its decomposition (Ortega et al. 2013; Kayaalti et al. 2016).

Guiyu, Guangdong Province, known as the “World Electronic Waste Disposal Station,” has a greater than 20-year history of e-waste recycling. Every year, this town disposes of millions of tons of e-waste, electrical appliances, and plastics from various countries, such as the United States, Germany, Japan, and Korea. Local workers use simple and rudimentary disassembly methods to handle waste, such as combustion and acid washing. Without any further treatment, acid washing wastewater that has heavy metal concentrations seriously exceeding the standards is then discharged into nearby streams, which are often used for irrigation or even drunk directly by local residents. The leftover waste is incinerated. It is difficult to completely break down e-waste; hence, toxic substances, such as lead, cadmium, polychlorinated biphenyls, carbon chloride and brominated flame retardants, inevitably persist in the environment. Huo et al. reported that the soil lead and cadmium levels in Guiyu were 2.32 and 4.34 times, while the ratios for dust sample were 4.10 and 3.18 times higher than a non-e-waste area (Yekeen et al. 2016). In comparison to the reservoir outside of Guiyu, Wang et al. found that the sediments of Lianjiang and Nanyang River in Guiyu were more enriched with dissolved lead, cadmium, cuprum, nikel and zinc (Wang et al. 2009). Rice is a kind of major staple food in China and its quality is critical to human health. According to Fu et al., the mean level of lead in polished rice reached 0.69 mg/kg, 3.5-fold higher than the maximum allowable concentration (MAC) (0.20 mg/kg) by the safety criteria for milled rice (NY5115-2002) (Fu et al. 2008). Long-term lead-containing food intake and environmental exposure to excessive lead can cause lead poisoning. Studies have shown that lead exposure in Guiyu had detrimental effects on the skeletal growth and neurobehavioural development of local children (Yang et al. 2013; Liu et al. 2011). Moreover, lead poisoning and other heavy metal poisoning are important factors leading to anaemia in children.

There are some reports related to lead exposure and anaemia. A study showed that blood lead and blood cadmium had an effect on Hb level. Blood lead and blood cadmium may interact with Hb (Chen et al. 2015). Kutllovci-Zogaj et al. used Spearman correlation analysis to analyse the relationship between BLL and Hb level in children in Mitrovica and found that there was a moderate correlation between BLL and Hb level (Kutllovci-Zogaj et al. 2014). However, previous studies did not exclude the influence of common nutritional and genetic factors in the impact of lead exposure on Hb. It is well known that nutrition and some genetic factors have a great influence on the haemoglobin of preschool children. Hence, we conducted an investigation to address the issue of e-waste and its potential effects on Hb synthesis in children on the premise of excluding common nutritional and genetic factors.

## Methods

### Study population

This cross-sectional study included 222 children (boys: 126; girls: 96) aged 3–6 years from two kindergartens in Guiyu, an e-waste disposal area (exposed group). In addition, 204 children (boys: 112; girls: 92) aged 3–6 years were recruited from three kindergartens in the Haojiang District (mainly engaged in agriculture and fishery, approximately 50 km from Guiyu) (control group) in 2014. This district does not conduct e-waste disassembly operations. All participants fulfilled the inclusion criteria as determined by a questionnaire survey and laboratory tests: (1) they lived in the study area for at least 3 years; (2) they had no serious haematological system disease in the past, such as aplastic anaemia and leukaemia; (3) they had no history of taking iron, folic acid or vitamin B_12_ in the past three months; and (4) thalassemia was excluded by Hb electrophoresis screening. Medical examinations were conducted with the permission of children and the written permission of the children’s guardians and the approval of the Ethics Committee of the Medical College of Shantou University.

### Sample collection and biochemical measurements

Care was taken to ensure that the venepuncture sites (routinely the back of the child’s hand) were completely sterilized, and 5 ml of venous blood was collected by a professional nurse. One millilitre of whole blood was immediately tested for Hb by the Hb cyanide (HiCN) method. One millilitre of whole blood was centrifuged at 5000 rpm for 5 min for Hb electrophoresis screening. Serum was obtained from two millilitre of whole blood and centrifuged at 3000 rpm for 15 min to determine ferritin, folic acid and vitamin B_12_ levels. Hb level was measured using an automatic LH-780 haematology analyser (Beckmann, Germany). Hb electrophoresis was done using agarose gel zone electrophoresis (PN1210 electrophoresis apparatus, Sevilla, France). Ferritin, folic acid, and vitamin 12 levels were determined via chemiluminescence (Advia Centaur XP automatic immunoassay of Siemens, Germany). Because children in both areas did not get their blood drawn at the same time, to reduce the detection error, the remaining 1 ml of blood was placed in a 2-ml vacuum blood vessel containing EDTA (40 U) anticoagulant, and each blood sample was stored at −20 °C to detect the BLL. The blood collection and sample management processes were carried out by professional staff, and all laboratory indicators adhered to standard reference materials.

### Measurement of BLLs

Before analyses, 100 μL of sampled blood was added to 900 μl of 0.5% nitric acid. This was then vortexed and digested at room temperature for 10 min. The resulting mixture was then used for the determination of lead levels.

Lead levels were determined using graphite furnace atomic absorption spectrophotometry (Zenit 650, Analytik Jena, Jena, Germany), which was done with an MPE60 autosampler (Analytik Jena, Jena, Germany) with an injection volume set at 20 μL. The main parameters used for the detection of lead levels were a wavelength of 283.3 nm; a lamp current of 4.0 mA; a slit width of 0.8 nm; drying at 90 °C, 105 °C, and 120 °C; ashing at 600 °C; and atomization at 1500 °C. The standard calibration curve was plotted using the six working standard solutions, which were prepared from a stock lead standard solution diluted with nitric acid, to which a matrix modifier mixed with human blood was added. The linear correlation coefficient of the lead standard calibration curve was 0.9920. The accuracy of the method was controlled by the use of recoveries between 96 and 108% (Rui-biao et al. 2015).

### Diagnostic criteria

According to the World Health Organization/UNICEF 2001 standard, anaemia is represented by Hb under 110 g/L in those aged 6–59 months or a Hb less than 115 g/L (level) in those aged between 6 and 11 years old (WHO 2011 ). Child BLLs were subdivided into low BLLs and high BLLs groups at 10.0 µg/dL, in reference to the U. S. Center for Disease Control and Prevention (CDC) (Brown 2007). This is then amended to 5.0 µg/dL (Betts 2012). A ferritin level of < 16 µg/L indicates iron deficiency (Powers and Buchanan 2014). Beta-thalassemia occurs when HBA_2_ is > 3.5%. Alpha poverty is diagnosed when HBA_2_ is < 2.5% or via abnormal HBH (HBBART) (Brancaleoni et al. 2016). A plasma folate level of 3 ng/mL indicates folate deficiency. A plasma Vitamin B_12_ level of 200 pg/mL indicates folate deficiency (Finglas 2000).

### Questionnaire investigation

The parents of the children examined were asked to complete a questionnaire regarding the socio-demographic characteristics of the children. The contents of the questionnaire mainly included the child’s birthplace, health status, surroundings, family economic conditions, and parental education level. Three classifications of total family income were defined according to the following levels: < 1999 Chinese *yuan* (333.2 US *dollars*, 1.0 US *dollar* ≈ 6.0 Chinese *yuan* in 2014) per month equated to low income, 2000–4999 Chinese *yuan* (333.3—833.2 US *dollars*) per month equated to middle income, and 5000 Chinese *yuan* (833.3 US *dollar*) per month equated to high-income families.

### Statistical analysis

Data entry and statistical analysis were performed using SPSS 15.0 (SPSS Inc., Chicago, IL, USA). Data distribution was tested for normality with the Kolmogorov–Smirnov test. Hb level was normally distributed, and BLL, folate, vitamin B_12_ and general characteristics were skewed. Data are expressed as arithmetic mean ± standard deviation (SD), median (25th–75th percentile), or number (percentage), depending on their distribution. Comparisons between two independent samples were performed using the *t* test or Mann–Whitney *U* *test.* Three subgroups were created according to BLL (Group A: < 5.0 µg/dL; Group B: 5.0–9.9 µg/dL; Group C: ≥ 10.0 µg/dL) and compared by using one-way ANOVA. Categorical data were compared using the *χ*^2^ test. The significance level was *P* < 0.05.

## Results

### General characteristics and nutritional status of children in the two towns

The general characteristics (including age, sex, family economic condition and parental education level) and nutritional status (including ferritin, folate and vitamin B_12_) of the children in two towns are shown in Table 1. There was no significant difference in age, sex, family income, or parents’ education level between the two study groups (*P* > 0.05). All children were excluded from thalassemia by Hb electrophoresis. There was no significant difference between groups in the level of ferritin, folic acid, or vitamin B_12_ in children with anaemia (*P* > 0.05).

**Table 1:** Comparison of overall characteristics and nutritional status of the children in the two towns

	Exposed group (*N* = 222)	Control group (*N* = 204)	*P* value
Age in months	61.8 ± 9.0	60.0 ± 10.2	0.07^a^
Boys, *n* (%)	126 (56.8)	112 (54.9)	0.70^c^
Family income more than 2000 yuan per month, *n* (%)	136 (61.3)	128 (62.7)	0.29^c^
Fathers with an education level of senior high school and above, *n* (%)	166 (74.8)	160 (78.4)	0.37^c^
Mothers with an education level of senior high school and above, *n* (%)	180 (81.1)	170 (83.3)	0.54^c^
Ferritin, µg/L	49.9 (35.1–68.4)	49.7 (36.1–66.2)	0.87^b^
Folate, ng/mL	7.3 (5.4–10.0)	8.8 (6.8–11.2)	0.98^b^
Vitamin B_12_, pg/mL	720.0 (577.0–851.3)	645.0 (491.0–824.0)	0.06^b^

Values are arithmetic mean ± SD, median (25th-75th percentile) and number (percentage)

^a^*P* value calculated by *t* test

^b^*P* value calculated by the Mann–Whitney *U* test

^c^*P* value calculated using the *χ*^2^ test

Unit measurement: 1 mg/L = 1000 µg/L (µg/L—microgram/litre); 1 mg/mL = 1,000,000 ng/mL (ng/ml -nanogram/millilitre); 0.001 ng/ml = 1 pg/mL (picogram/millilitre).

### BLLs of children in the two towns

There was a significant difference in BLL between the two groups. The average BLL in the exposed group was higher than in the control group (*P* < 0.01). The risk of children with BLLs above 10 µg/dL in the exposed group was significantly higher than that in the control group (*P* < 0.01), while no difference in the two groups with BLLs between 5.0 and 9.9 µg/dL (*P* > 0.05). The data are shown in Table 2.

**Table 2:** Comparison of the average BLLs and rate of high BLLs between groups

	BLLs, µg/dL					BLLs 5.0–9.9 µg/dL				BLLs ≥ 10.0 µg/dL			
	*N*	median	25th-75th percentile	*Z* value	*P* value	*n*	Rate (%)	*χ*^2^ value	*P* value	*n*	Rate (%)	*χ*^2^ value	*P* value
Exposed group	222	8.5	6.6–10.9	− 8.71	0.00^a^	115	51.8	0.41	0.52^b^	74	33.3	19.99	0.00^b^
Control group	204	6.0	4.8–7.9			112	54.9			30	14.7		

^a^*P* value calculated by the Mann–Whitney *U* test

^b^*P* value calculated using the *χ*^2^ test

### Effects of different doses of BLLs on Hb concentration in the two towns

The study participants were divided into three subgroups on the basis of their BLLs, including Group A (BLL < 5.0 µg/dL), Group B (BLL 5.0–9.9 µg/dL), and Group C (BLL ≥ 10.0 µg/dL). Table 3 shows that there was no significant difference in the Hb levels between the three subgroups of the control group (*P* > 0.05). However, a significant difference was observed in the Hb levels between the three subgroups of the exposed group by one-way ANOVA, with a tendency for a decrease in Hb level with rising BLL in the exposed group (*P* = 0.03) but not in the control group (*P* = 0.14). In addition, the Hb levels of group B and group C were also significantly lower in the exposed group than in the control group (Group B: 122.6 ± 9.5 g/L versus 125.8 ± 8.2 g/L, *P* = 0.01; Group C: 120.3 ± 7.3 g/L versus 123.6 ± 8.3 g/L, *P* = 0.03).

**Table 3:** Effects of BLL on Hb concentration in the two towns

BLLs	Exposed group (*N* = 222)		Control group (*N* = 204)		*T* value	*P* value^a^
	*n*	Hb ($\overline{x}$ ± s, g/L)	*n*	Hb ($\overline{x}$ ± s, g/L)		
< 5.0 µg/dL (Group A)	33	124.9 ± 8.2	62	126.9 ± 8.5	− 1.14	0.26
5.0–9.9 µg/dL (Group B)	115	122.6 ± 9.5	112	125.8 ± 8.2	− 2.54	0.01
≥ 10.0 µg/dL (Group C)	74	120.3 ± 7.3	30	123.6 ± 8.3	− 2.26	0.03
*F* value		3.52		1.98		
*P* value^b^		0.03		0.14		

^a^*P* value calculated by *t* test

^b^*P* value calculated by one-way ANOVA

### Comparison of influencing factors of anaemia among children in the two towns

As shown in Table 4, the prevalence of anaemia associated with BLLs above 10 µg/dL and between 5.0 and 9.9 µg/dL were both significantly higher in the exposed group than in the control group (4.0% vs. 0.5%, 5.4% vs. 1.5%, respectively, both *P* < 0.05). There was no significant difference between the exposed group and the control group in the prevalence of anaemia associated with iron deficiency (2.3% vs. 2.9%, *P* > 0.05). Furthermore, the prevalence of anaemia in children with low BLLs (< 5.0 µg/dL) and with non-iron deficiency was also no significant difference between the two groups (1.4% vs. 1.5%, *P* > 0.05).

**Table 4:** Analysis of influencing factors of anaemia^a^ in children in an e-waste dismantling area

	Exposed group (*N* = 222)		Control group (*N* = 204)			
	Anaemia, n	Anaemia, %	Anaemia,n	Anaemia, %	*χ*^2^ value	*P* value
BLLs > 10 µg/dL	9	4.0	1	0.5	4.44	0.03
BLLs 5.0–9.9 µg/dL	12	5.4	3	1.5	4.85	0.03
Iron deficiency^b^	5	2.3	6	2.9	0.20	0.65
BLLs < 5 µg/dL and non-iron deficiency	3	1.4	3	1.5	0.00	1.00

^a^Haemoglobin (Hb) < 11.0 g/dL for children between 6 and 59 months and Hb < 11.5 g/dL for children between 6 and 11 years old

^b^Ferritin level < 16 ng/mL

## Discussion

Worldwide, the main reason for decreased Hb synthesis in children is iron deficiency (WHO 2001). Other causes include folic acid and/or vitamin B_12_ deficiency and thalassemia (Madu and Ughasoro 2017). To explore the effect of e-waste pollution on Hb synthesis in children, it is necessary to exclude these common nutritional and genetic factors. Out of this concern, normal levels of ferritin, folic acid, and vitamin B_12_ were required for our study, and there was no significant discrepancy in these levels between groups (*P *> 0.05). There is a relatively high incidence of thalassemia in Guangdong Province, as one study found an incidence of approximately 8.53% (Xu et al. 2004). To exclude any effect of thalassemia, we confirmed that no children who participated in our study had thalassemia by Hb electrophoresis screening.

It was necessary to understand the general characteristics (including social demography and cultural information) and nutritional status of children in the two regions. Families with higher maternal education may have better economic resources or engage in behaviours protective of child health, such as iron supplementation. The results showed that there was no significant difference in age, sex, family income, or parents’ education level distribution between the two study groups (*P* > 0.05). This means that families in both places were unlikely to have differences in the rate of insufficient dietary intake due to economic difficulties.

As shown in Table 2, the average BLL in the exposed group was higher than that in the control group (*P* < 0.01). In addition, the risk of children with BLLs above 10 µg/dL in the exposed group was significantly higher than that in the control group (*P* < 0.01). These results indicate that e-waste pollution had an impact on BLL in the study population. Whether lead can inhibit Hb synthesis is closely related to the child's lead load level. Research by Chen and Kutllovci-Zogaj showed that blood lead could interfere with Hb synthesis (Chen et al. 2015; Kutllovci-Zogaj et al. 2014). The United States Centers for Disease Control (CDC) has recommended that BLL can be used for the assessment of the lead load in the body and the degree of lead poisoning. At present, a BLL of 20–40 µg/dL is considered the critical range at which normal Hb synthesis is affected. When the BLL is 40–60 µg/dL or > 60 µg/dL, Hb synthesis will be inhibited, and there is an 18% to 40% probability of anaemia at the two former levels (Schwartz et al. 1990). In this study (Table 2), the majority of the children in both the exposed and the control groups had BLLs of less than 20 µg/dL. This indicates that low blood lead concentrations may also inhibit Hb synthesis. According to the NHANES III survey in the United States, Hb synthesis can begin to be inhibited even at a BLL of 5.0–9.9 µg/dL (Bernard and McGeehin 2003). Data suggested that lead-induced anaemia is mediated by the inhibition of Hb synthesis (Osterode et al. 1999).

This study showed that Hb levels in the exposed group decreased significantly with increasing blood lead load, but not in the control group (Table 3). Moreover, the prevalence of anaemia in children with high BLLs (≥ 5.0 µg/dL) in the exposed group was significantly higher than that in the control group (Table 4). This indicated that lead exposure in Guiyu is different from that in the non-e-waste disposal area, and there may be coexisting factors that enhance lead blood toxicity. In addition to lead, e-waste contains more than 700 substances, 50% of which may endanger human health, such as cadmium, mercury, hexavalent chromium, polyethylene, polystyrene, brominated flame retardants, and surface coatings (Davis and Heart 2018). It has been reported that 58 kg of mercury, 24.6 kg of cadmium 340.5 kg of arsenic, and several other substances can be separated from 1 ton of randomly collected electronic cards (Jin-qiu et al. 2007). These toxic substances may interact with lead or cause lead to inhibit Hb more strongly than when acting alone, even in a mild elevated BLL (5.0–9.9 µg/dL). Our previous study in this investigation group showed that there was a negative correlation between blood cadmium and Hb level in children in Guiyu (Rui-biao et al. 2015), which is consistent with other findings that the Hb level in the high-cadmium group was lower than that in the low-cadmium group (Jie-dan et al. 2010). Other substances are contained in e-waste, such as aluminium and benzene derivatives, have also been reported to inhibit Hb synthesis (Lin et al. 2013; Ghosh et al. 2017; Snyder 2000). In terms of heavy metal only, the analysis of river water samples and river sediment collected in Guiyu showed that these had high contents of silver, chromium, mercury nickel, copper, lead, zinc and other heavy metals. Chromium, mercury nickel, copper, lead, zinc and other heavy metals have common target organs and molecular targets, such as affecting the haematopoietic system of bone marrow, which would lead to anaemia. There are usually interactions between metals due to their similar chemical properties (Coby et al. 2007). Therefore, further study of the effects of other heavy metals on anaemia in children is required.

Studies have shown that lead can occupy iron ion sites in the intestinal mucosa or haematological system, thus increasing the absorption or reducing the excretion of lead. Therefore, with the increase in lead load in children, iron content will correspondingly decrease, which can lead to an increase in the incidence of iron deficiency anaemia (Conrad et al. 1992; Bradman et al. 2001). Unexpectedly, there was no significant difference in the incidence of iron deficiency anaemia between the exposed group and the control group (*P* = 0.65). The possible reasons might be that few children had BLLs > 20 µg/dL, and normal ferritin status does not necessarily indicate iron sufficiency because ferritin is an acute-phase reactant and may be elevated by infection or inflammatory disease (Caterina and Maria 2008). Thus, some iron-deficient children may have been misclassified as iron-replete on the basis of the ferritin level, which would bias the data.

However, the study also has limitations that may bias the results. First, our study corrected the factors that may affect hemoglobin synthesis, such as folic acid and B_12_. But there are many factors affecting anemia, such as vitamin A deficiency and zinc deficiency, which will affect the synthesis and metabolism of hemoglobin. Second, in this study, hemoglobin screening was used to exclude children with thalassemia, but it did not exclude some diseases that may also cause anemia such as chronic infections and kidney diseases. Third, our study did not measure erythropoietin, hepcidin, transferrin receptor, transferrin and other iron metabolism parameters. Finally, the sample size of the study subjects was small.

In conclusion, lead exposure (excluding the effects of common nutritional factors and thalassemia) more significantly inhibits Hb synthesis in children who live in e-waste dismantling areas than in those who live in non-e-waste dismantling areas. Other toxins released from e-waste may also contribute to the inhibition of Hb synthesis and may even lead to anaemia in local children. Further investigations are needed to expand the sample size and provide evidence for the development of relevant protective measures.

## Abbreviations

E-waste: Electronic waste
Hb: Haemoglobin
BLL: Blood lead level
ALAD: Aminoethyl propionate dehydrase
ALA: Aminoethyl propionate
SD: Standard deviation
